# Is cancer risk reduced in multiple sclerosis? Results from a tertiary referral center

**DOI:** 10.55730/1300-0144.5660

**Published:** 2023-02-27

**Authors:** Yasemin BAHAR, Egemen YILDIZ, Abdul Rasheed BAHAR, Rana KARABUDAK

**Affiliations:** 1 Department of Medicine, Faculty of Medicine, Hacettepe University, Ankara, Turkiye; 2 Department of Emergency Medicine, Sultan 2. Abdulhamid Han Training and Research Hospital, University of Health Sciences, İstanbul, Turkiye; 3 Department of Neurology, Faculty of Medicine, Hacettepe University, Ankara, Turkiye

**Keywords:** Multiple sclerosis, cancer, risk factors, immune system

## Abstract

**Background/aim:**

Multiple sclerosis (MS) patients may be protected against cancer because of increased immune surveillance. However, aberrant T/B cell functioning in MS may increase the risk of cancer. We aimed to compare the frequency of cancer among patients with MS with an appropriate control group matched by the variables such as age, gender, tobacco smoking history, body mass index (BMI), and family history of cancer.

**Materials and methods:**

The MS patients who were registered and followed up at the MS Center in Hacettepe University Hospitals and appropriately matched with controls were included. A self-administered questionnaire with links to the online survey was delivered.

**Results:**

Overall, 1037 responses out of 2074 in MS patients and 506 responses out of 1500 control group were included. Fourteen (1.35%) of MS patients and 18 (3.6%) of the controls were diagnosed with cancer. The odds ratio of having cancer in patients with MS compared to the control group was 0.389 (95% CI = 0.161–0.940, p < 0.05).

**Conclusion:**

There was no statistically significant difference in age, gender, tobacco smoking, and BMI between the groups after propensity score matching. The odds of having cancer were lower in our MS patients compared to the controls. The autoimmune changes responsible for the pathogenesis of MS may be responsible for the decrease in cancer risk.

## 1. Introduction

Multiple sclerosis (MS) is the most common autoimmune inflammatory demyelinating disease of the central nervous system and the leading cause of nontraumatic permanent disability in young adults [1,2]. The prevalence of MS varies significantly across the world, with an overall female predominance. Although the exact etiology of MS remains unknown, the most widely accepted hypothesis is that MS begins as an inflammatory immune-mediated process characterized by autoreactive lymphocytes [3, 4]. In addition, certain environmental factors may play an important role in determining the risk of multiple sclerosis in genetically predisposed individuals [5].

The immune system, particularly regulatory T cells, is thought to play a major role in the pathogenesis of both cancer and MS [6, 7]. Although the activation of the immune system in MS may be associated with increased immune surveillance, the chronic inflammation and the chronic use of some immunosuppressive (IS) or immunomodulatory (IM) agents for the treatment of MS may be associated with an increased risk of cancer as well [8].

Studies regarding the risk of cancer in MS have been associated with conflicting results. While most studies suggested no association or a small decrease in the prevalence and incidence of cancer among patients with MS [9–13], some resulted in an increased risk of cancer among MS patients [14,15].

This study aims to compare the frequency of cancer among MS patients with an appropriate control group matched by the variables that may affect the relationship between MS and cancer such as age, gender, tobacco smoking history, body mass index (BMI), and family history of cancer.

## 2. Materials and methods

### 2.1. Subjects

Patients who were followed up at the Neurology Department of Hacettepe University Hospitals and with the ICD-10 code for MS (G35) on the local electronic database in the last ten years, were invited to participate in the study. The specialized MS clinic at Hacettepe University serves as one of the main referral centers for MS patients from all regions of Turkey and has been active since 1995. The diagnosis of MS in the invited patients was confirmed by an MS specialist. Patients with MS in our study also included Clinically Isolated Syndrome and Radiological Isolated Syndrome.

Patients with MS who were followed up at the Neurology Department of Hacettepe University Hospitals with the ICD-10 code for MS (G35) on the local electronic database in the last ten years and agreed to participate in the study and were over the age of 18 were included in the study. The control group consisted of randomly selected people who were older than 18 years to whom the online form of the questionnaire was sent via mailing lists of several universities, corporations within different regions, and social media platforms and groups reached by the authors. The exclusion criteria for the control group were being under 18, having MS disease, and receiving any IM or IS therapies for chronic illness. To avoid selection bias, we avoided specific statements in the cover letter of the questionnaire which could attract only the relevant cases such as cancer to the study. We stated that the research was conducted on a voluntary basis. The study was approved by the appropriate institutional review board.

### 2.2. Survey questionnaire

A total of 2091 patients were selected from a local electronic database, and 2074 of them were confirmed to have MS after a careful review by a neurologist. A self-administered questionnaire was then delivered to these patients by a phone call or a short message that contained the links to the online survey between February 1st, 2019, and March 1st, 2020. The online form of the questionnaire was also sent to the rest of the population via mailing lists of several universities, corporations within different regions, and social media platforms and groups reached by the authors to reach a more diverse representation. To avoid selection bias, people visiting the hospital for other purposes were not used as a control group. Questionnaires were secondarily anonymized, and only one person had access to the corresponding list. Figure shows our algorithm for participant selection and grouping.

In addition to the questionnaire, informed consent was sent to all participants. For the participants, giving informed verbal consent by phone call and approving informed consent to participate in the online survey was considered consent. The participants completed the survey on their own and did not receive any help from members of the research team.

MS patients responded to the questions regarding their gender, date of birth, history of cancer and cancer lesions, family history of cancer, smoking history (daily use during lifetime to calculate pack-year), history of alcohol consumption, height and weight to calculate BMI, regular physical activity and treatments received for MS. Patients with a history of cancer had to indicate the year of diagnosis, type of cancer, the medical doctor who diagnosed cancer or the surgeon who operated on cancer, and the hospital where the patient with cancer was diagnosed to permit retrieval of confirmation of cancer diagnosis. To reduce the risk of under-reporting, questions regarding cancer or a precancerous lesion were followed by a list of 20 different potential cancer sites to make reporting easier. It is noteworthy that nonmelanoma skin cancers were not included in the list.

MS patients also had to declare the date of their first MS signs, the date of MS diagnosis, and the name of the DMT taken during the disease course. The control group was asked the same questions except for the questions related to MS. We included in our analysis an oncological diagnosis that occurred after the initial symptoms of MS.

### 2.3. Sample size calculation and statistical analysis

Using data from previous studies, the required number of participants for sampling was calculated as 503 subjects in each group (MS patients and controls), with a type 1(α) error rate of 5% and statistical power of 93%.

All statistical analyses were performed using IBM SPSS Statistics 25.0 (IBM Corp., Armonk, NY) program for Windows. All continuous data reported in this study are expressed as median [interquartile range (IQR), and range] for each value (assumption of normality was assessed using the Kolmogorov-Smirnov, and the Shapiro–Wilk test). Continuous variables were compared between the two groups using the Mann-Whitney U test, and the chi-square test was used for the categorical variables.

Since the baseline characteristics and factors that can affect the frequency of cancer in MS patients differ from control subjects, propensity score matching analysis using the nearest neighbor pair matching method with a caliper width of 0.2 was performed [16]. Stratification on the propensity score involves grouping subjects into mutually exclusive groupings based on their estimated propensity score. The calculated propensity score is used to rank the subjects. Then, depending on previously established predicted propensity score thresholds, subjects are separated into subsets. Consequently, the distribution of measured baseline covariates between cases and controls within the same stratum will be roughly identical when the propensity score has been properly set. The covariates included in the propensity score model were gender, age, tobacco smoking history, BMI, and family history of cancer. MatchIt package [17] (R software) was used for this analysis. Logistic regression analysis was performed to determine the independent predictors. Hosmer-Lemeshow goodness of fit statistics was used to assess model fit. All analyses were evaluated at the 95% confidence interval, and significance was evaluated at the p < 0.05 level.

## 3. Results

Overall, 1037 responses out of 2074 MS patients were included. The results of the patient survey were verified from the database. It was concluded that the reasons for the 50% participation rate include; not updating contact information, death, no participation due to low education level, and unavailability. The median age of MS patients was 37 years [IQR 15 years, range 19 to 74 years]. At the same time, an online questionnaire was delivered to 1500 people without MS disease in the control group. Five hundred and six people out of 618 who fulfilled the inclusion criteria and responded to our online survey were included in the study, thus, the response rate for the control group was calculated to be 41.2%. The median age of the control group was 36 years (IQR 14 years, range 18 to 71 years). Seven hundred and thirteen (68.8%) of MS patients and 322 (63.6%) of the control group included in the study were female. The median number of years after MS diagnosis was 8 (IQR 3 years, range 1 to 40 years).

BMI, family history of cancer, tobacco smoking history, regular alcohol consumption, regular physical activity status, pack-year values of smokers, and the number of days with exercise for regular exercisers are shown in Table 1 for both MS patients and the control group. The MS patient group had a lower BMI value (p < 0.001). The percentage of people having a family history of cancer was lower for MS patients (p < 0.001). When MS patients and the control group were compared according to smoking status; it was found that the rate of smoking was higher in the MS patient group and they had a higher number of pack-years (p = 0.024, p < 0.001 respectively). The percentage of alcohol consumers was also higher for MS patients (p < 0.001 respectively).

Fourteen (1.35%) of MS patients and 18 (3.6%) of the control group included in the study were diagnosed with cancer. The most common ones were breast cancer (N: 10), cervix cancer (N: 5), and thyroid cancer (N: 4). Clinical and demographic characteristics of MS patients with a cancer diagnosis are shown in Table 2.

Ten (71.4%) of these MS patients had received disease-modifying treatments (DMT), and four of them had used more than one DMT. Interferon-beta (N: 7), glatiramer acetate (N: 5), dimethyl fumarate (N: 2), and ocrelizumab (N: 2) were used for the treatment of the MS patients who were later diagnosed with cancer.

The propensity score matching was performed for age, gender, tobacco smoking history, BMI, and family history of cancer. Then, 505 MS patients were matched with 505 control subjects. The characteristics of MS patients and controls after matching are shown in Table 3. After logistic regression analysis, it was found that the odds ratio of having cancer in patients with MS compared to the control group was 0.389 (95% CI = 0.161–0.940, p < 0.05).

## 4. Discussion

To our knowledge, our study was the first to investigate the cancer frequency among MS patients in Turkey and compare it with the matched control group. Our results demonstrated that the risk of getting cancer in MS patients was 61.1% of that of the control group. Age, gender, tobacco smoking history, regular alcohol use, regular exercise, BMI, and family history of cancer are all evaluated. Our study’s main strength is that it is the first to consider such a large number of cancer risk factors. Previous studies lacked information about confounding risk factors related to cancer such as BMI, family history, and smoking status, which may have an important effect on cancer frequency [18].

Some prior studies have resulted in a decreased cancer risk in MS patients, which is consistent with our findings [19–21]. The relationship between MS and cancer is complicated. While persistent inflammation and chronic immunosuppression increase the risk of cancer, autoimmune disorders such as MS may have enhanced immunosurveillance due to the activation of inflammatory cells, consequently decreasing the risk of cancer development. Additionally, since the incidence of cancer increases with age for the major cancers, survival could be biasing comparisons due to early death from MS [21]. Other factors which could explain the decreased cancer risk in this population include behavioral modification and the treatment of MS [8, 22].

Contrary to our findings, some studies have reported an increased risk or no difference in the risk of cancer frequency in patients with MS compared to the general population [21, 23]. In a prospective cohort study, Grytten and collaborators found an overall 14% increased risk of cancer among MS patients compared with population controls, especially in respiratory organs, urinary organs, and the central nervous system [24].

Breast cancer was the most common cancer type in our study, as one might predict in a population of middle-aged women. Similarly, the most common cancer among women according to the Turkish Ministry of Health, reported in 2017, was breast cancer.^1^ There is evidence of an increased risk of developing certain types of cancer in patients with MS, especially meningioma and cancers of the urinary organs [21]. Because there were an inadequate number of malignancies of each type for analysis, this study was unable to assess the risk for particular types of cancer. A family history of cancer is a significant risk factor for a variety of malignancies [25].

There is no reliable source that reflects the entire population that could be used to evaluate cancer statistics in Turkey. The 2017 Turkey Cancer Statistics Report^1^ included data from the cancer registries of 14 provinces and projected only 50% of the total population. Also, İstanbul’s cancer registry data, which includes approximately one-fifth of Turkey’s population, was not used and only the cancer registry records of 9 provinces were used in the Globocan 2020 data.^2^ Therefore, due to both the limited number of participants in our study and the limitations of Turkey’s cancer data in reflecting the total population, it is considered inappropriate to attempt to reach a conclusion by comparing the data.

The consanguinity rate among people living in Turkey is approximately %23 [26]. This fact may subsequently result in an increased familial cancer rate. We detected two cases of Lynch syndrome and a BRCA mutation in our MS cohort. Due to the proximity of the MS locus to the BRCA1 gene, this condition may lead to breast cancer and MS simultaneously [27].

Since the immune system is critical for recognizing and eliminating cancer cells, immunosuppression from DMTs may subsequently increase the risk of cancer development in MS patients. However, studies regarding the effect of DMTs on cancer have obtained contradictory results, with some resulting in an increased risk of cancer, while others have shown no increased cancer risk. This disparity is further complicated by the various types of DMTs used in patients with MS [20, 28–31]. A Danish nationwide cohort study also found that DMTs in Danish MS patients were neither accompanied by an increase in cancer incidence nor an increase in cancer-specific mortality [18]. Most of the MS patients with cancer in our study had received DMT and four of them had used more than one DMT. Interferon-beta was the most common DMT. Meta-analysis and further research are required to understand the effects of DMTs on cancer risk in MS patients.

Tobacco smoking and the use of alcohol are accepted risk factors for developing cancer [32]. After matching the propensity score according to smoking status; the remaining MS group had a lower cancer rate compared to the control group despite having a higher median of cigarette packs × years. Alcohol consumption data was not included in analyses since underreporting was suspected in a similar context in previous studies in the literature [33, 34]. In this study, propensity score matching was performed for age, gender, tobacco smoking history, BMI, and family history of cancer. This method’s primary goal is to generate an unbiased estimate of outcome measures in nonrandomized and observational studies that has been adjusted for the impact of specific confounding factors. The propensity score’s major advantage is its decrease in dimensions, which solves the problem of an insufficient number of sample instances in exact matching. In actual research, several factors that represent many dimensions must frequently be adjusted for. These dimensions are condensed into a single dimension using the propensity technique.

Our study is limited in investigating the effect of DMTs on cancer development in MS patients. Most of our MS patients who were diagnosed with cancer were using DMTs. Due to an insufficient number of MS patients diagnosed with cancer, it is difficult to deduce that the use of DMT may be a risk factor.

We have tried the best we can with a basic survey aiming to see the cancer risk profile in our MS patients’ registry. However, some shortcomings need to be studied and clarified further. One limitation of our study is that there were nonresponders among MS patients and the control group. Nonresponse bias can skew conclusions generated from questionnaire surveys across people, countries, and topics. Nonresponders were more likely to be male, less educated, single or divorced, or immigrants in studies of general population samples in North America and Europe [35,36]. Because particular subpopulations are underrepresented in surveys, the effects and estimations in the results may be underestimated or exaggerated. We also believe that there is a need for a nationwide MS registry and a cancer database to support further research.

Self-reporting of cancer might be considered as a bias. Although the conditions for screening for cancer in both MS patients and the control group were the same. To reduce the risk of under-reporting, questions regarding cancer or a precancerous lesion were followed by a list of 20 different potential cancer sites to make reporting easier. Also, Navarro et al. [37] and Cowdery et al. [38] found moderate concordance between self-reported cancer history and state registry data for any cancer, with excellent specificity and negative predictive value. It is also stated in that study that self-reported cancer data was adequate for detecting a cancer history, but it was less reliable in identifying the history of particular cancer types indicated in registry-based data. In addition to that, the number of cancer cases was insufficient to draw conclusions for each subtype of cancer. Another limitation of our study is that we have not evaluated chronic diseases in our study. As certain chronic diseases predispose to cancer, this might be considered a potential source of bias [39].

Case-control studies may be prone to recall bias (e.g., alcohol consumption, physical activity). Because these studies are retrospective in design, individuals with a disease may be more likely than those without a disease at the time of data collection to report risk factors from the past [40]. Also, social desirability response bias can affect results in self-reported research [41]. In this study, for alcohol consumption data, we predicted that bias and did not use it in further analyses.

In conclusion, there was no statistically significant difference in age, gender, tobacco smoking, and BMI between the groups after propensity score matching. Breast cancer was the most common cancer type in our study, as one might predict in a population of middle-aged women. The odds of having cancer were decreased in our MS patients. The autoimmune changes responsible for the pathogenesis of MS may be responsible fora decrease in cancer risk. Although the results of our study revealed a decreased frequency of cancer in MS patients compared to the control population, it should neither affect the quality of routine cancer screening programs nor cause any misinterpretation in this group. We are aware of and concerned about the impact of this matter. Although the number of patients and controls needs to be increased, our study highlights the MS-Cancer profile in Turkish MS patients. Based on this substantial number of participants, this study contributes to the current cancer frequency data in patients with MS, and we are encouraged to continue with more comprehensive epidemiological multicenter studies.

## Figures and Tables

**Figure:**
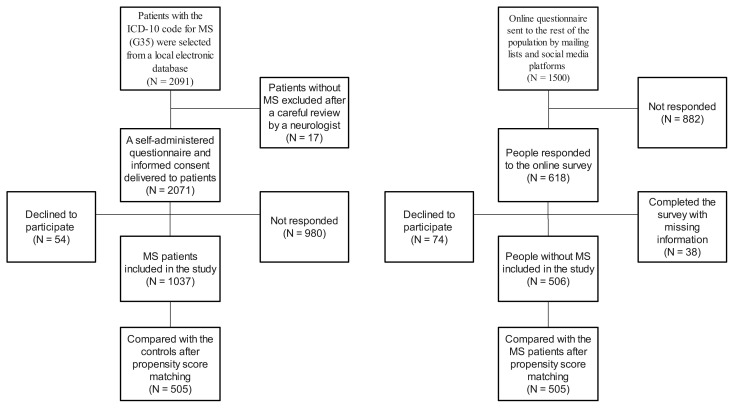
Flowchart of participant selection and information collection.

**Table 1:** Clinical and demographic characteristics of the study population.

	MS patients (n = 1037)	Control group (n = 506)	P-value
Age (median, IQR, min–max)	37, 15, 19–74	36, 14, 18–71	0.028ƚ
Gender (female: n, %/male: n, %)	713, 68.8%/324, 31.2%	322, 63.6%/184, 36.4%	0.045*
BMI (median, IQR, min–max)	23.95, 5.54, 14.20–43.37	24.91, 5.63, 15.24–54.43	<0.001ƚ
Family history of cancer (n, %)	467, 45.0%	274, 54.2%	<0.001*
Regular physical activity (n, %)	509, 49.1%	261, 51.6%	0.357*
Number of days with exercise (median, IQR, min–max)	3, 3, 1–7	3, 2, 1–7	<0.001*
Tobacco smoking history (n, %)	512, 49.4%	219, 43.3%	0.024 *
Pack-year of smokers (median, IQR, min–max)	10.0, 15.0, 0.01–88.0	6.0, 7.0, 0.1–69.0	<0.001ƚ
Alcohol consumption (n, %)	219, 21.1%	40, 7.9%	
Years after MS diagnosis (median, IQR, min–max)	8, 3, 1–40		
Cancer diagnosis (n, %)	14, 1.35%	18, 3.6%	

ƚ Mann-Whitney U test is used to compare differences.

* Chi-square test used for analysis.

BMI, body mass index; IQR, interquartile range; MS, multiple sclerosis.

**Table 2:** Clinical and demographic characteristics of MS patients with a cancer diagnosis.

	MS Patients (n = 14)
Age (median, IQR, min–max)	46, 20, 24–66
Gender (female: n, %/male: n, %)	12, 85.7%/2, 14.3%
BMI (median, IQR, min–max)	27.28, 6.55, 19.53–38.67
Family history of cancer (n, %)	9, 64.3%
Regular physical activity (n, %)	4, 28.6%
Number of days with exercise (median, IQR, min–max)	2, 2, 2–4
Tobacco smoking history (n, %)	8, 57.1%
Pack-year of smokers (median, IQR, min–max)	15, 18.25, 1.75–35
Alcohol consumption (n, %)	1, 7.1%
Years after MS diagnosis (median, IQR, min–max)	14, 12, 5–40
DMT history (n, %)	10, 71.4%
Interferon-beta (N: 7), months (median, min–max)	48, 12–132
Glatiramer acetate (N: 5), months (median, min–max)	40, 18–120
Dimethyl fumarate (N: 2), months (median, min–max)	36, 24–48
Ocrelizumab (N: 2), months (median, min–max)	18, 12–24

BMI, body mass index; IQR, interquartile range; MS, multiple sclerosis.

**Table 3:** The characteristics of MS patients and controls after propensity score matching.

	MS patients (n = 505)	Control group (n = 505)	P-value
Age (median, IQR, min–max)	37, 14, 20–71	36, 14, 18–71	0.115ƚ
Gender (female: n, %/male: n, %)	336, 66.5%/169, 33.5%	322, 63.8%/183, 36.2%	0.355*
BMI (median, IQR, min–max)	24.49, 5.51, 16.59–43.37	24.90, 5.63, 15.24–45.71	0.309ƚ
Family history of cancer (n, %)	239, 47.3%	274, 54.3%	0.028*
Regular physical activity (n, %)	256, 50.7%	261, 51.7%	0.753*
Number of days with exercise (median, IQR, min–max)	3, 3, 1–7	3, 2, 1–7	0.001
Tobacco smoking history (n, %)	233, 46.1%	218, 43.2%	0.342*
Pack-year of smokers (median, IQR, min–max)	10.0, 15.63, 0.2–88.0	6.0, 7.0, 0.1–69.0	<0.001ƚ
Alcohol consumption (n, %)	113, 22.4%	40, 7.9%	<0.001ƚ
Years after MS diagnosis (median, IQR, min–max)	8, 5, 2–40		
Cancer diagnosis (n, %)	7, 1.4%	18, 3.6%	

ƚ Mann-Whitney U test is used to compare differences.

* Chi-square test used for analysis.

BMI, body mass index; IQR, interquartile range; MS, multiple sclerosis.

## Footnotes

1 Halk Sağlığı Genel Müdürlüğü (2017). Kanser İstatistikleri (in Turkish) [online]. Website https://hsgm.saglik.gov.tr/tr/kanser-istatistikleri/yillar/2017-turkiye-kanser-i-statistikleri.html [accessed 10 August 2022].

2 International Agency for Research on Cancer (2021). Population Fact Sheets: Turkey [online]. Website https://gco.iarc.fr/today/data/factsheets/populations/792-turkey-fact-sheets.pdf [accessed 10 August 2022].
